# Comparative microbiomic analysis of fecal microbiota associated with abdominal fat in ducks

**DOI:** 10.1016/j.psj.2025.105282

**Published:** 2025-05-10

**Authors:** Yangyang Shen, Yuhang Li, Jing Xiao, Jiawei Li, Yongfei Wu, Yan Wu, Hongbo Tang, Xinyan Fang, Lei Wang, Yujie Gong, Hao Chen, Xueming Yan

**Affiliations:** Key Laboratory of Natural Microbial Medicine Research of Jiangxi Province, College of Life Sciences, Jiangxi Science and Technology Normal University, Nanchang, 330013, China

**Keywords:** Ducks, Abdominal fat, Gut microbiome, 16S rDNA sequencing, Metagenomic sequencing

## Abstract

The gut microbiota, which features complex community structures, colonizes the duck intestine and plays a crucial role in metabolism, immune regulation, and meat quality. Gut-microbiota-regulated abdominal fat deposition is a key factor that affects the meat quality of livestock and poultry. We used 16S rDNA and metagenomic sequencing to investigate the microbial community characteristics of 187 fecal samples from 10 Chinese indigenous duck breeds (five breeds for each of the high/low abdominal fat categories). We explored the relationship between fecal microbiota and abdominal fat deposition. The α diversity of the fecal microbiome in high abdominal fat ducks (HAF) was higher than that in low abdominal fat ducks (LAF). The fecal microbiota and function were also significantly different. At the phylum level, Actinobacteria was significantly enriched in HAF, whereas *Proteobacteria, Candidatus, Saccharibacteria,* and *Fusobacteria* were abundant in LAF. At the genus level, *Lactobacillus, Alistipes, Corynebacterium,* and *Lachnoclostridium* were more abundant in HAF than in LAF. The *Streptococcus, Campylobacter, Helicobacter, Enterobacter, Gallibacterium,* and *Escherichia* genera were significantly enriched in LAF. Microbial functional analysis indicated that the HAF fecal microbiota was mainly involved in carbohydrate, nucleotide, lipid, amino acid, terpenoids, polyketides, and xenobiotic metabolism. In addition, bacteria related to signal transduction, cofactor and vitamin metabolism, and infectious disease were enriched in LAF. This study revealed the relationship between gut microbiota and abdominal fat deposition in ducks. Our findings lay a foundation for the abdominal fat deposition mechanism in ducks and provide a reference for Chinese indigenous duck husbandry.

## Introduction

China is a major country for poultry production and consumption, and ducks are also important agricultural animals (Huang, et al., 2013a). Chinese indigenous ducks have rich species diversity, excellent stress resistance, roughage tolerance, and unique meat quality and are usually precious poultry genetic resources (Zhang, et al., 2018). Due to the lack of conservation measures, Chinese indigenous ducks have rapidly declined, resulting in the loss of genetic resources and characteristics.

Duck is a major source of quality meat and rich protein in the poultry industry (Huang, et al., 2013b). Notably, meat quality has always been important to consumers (Joo, et al., 2013). Therefore, the role of the gut microbiome on meat quality-related traits has gained substantial interest (Muinos-Buhl, et al., 2018). Fat deposition may directly affect the poultry performance, meat flavor, feed conversion ratio, and even the economic benefits of scale production. In animals, fat deposition, a complex biological process, is regulated by many factors such as genetics, nutrition, and especially the gut microbiome, which is well-associated with lipid accumulation (Chen, et al., 2022). Notably, obesity is highly correlated with the gut microbiota profile (Portune, et al., 2017). Since the gut microbiome is closely related to meat quality, excessive lipid accumulation and high oxidative stress present a serious health and economic issue in the poultry industry (Zhao, et al., 2022).

Gut microbiota involved in the regulation of host nutrient metabolism, immune response, intestinal development, and health (Cerf-Bensussan and Gaboriau-Routhiau, 2010) can decompose dietary fiber that the host cannot digest into monosaccharides and short-chain fatty acids, which are in turn absorbed, used, and converted into fat by the host (Pryde, et al., 2002). Storage in fat cells leads to abdominal lipid deposition (Backhed, et al., 2004; Krause, et al., 2020). Notably, the composition and structure of the gut microbial community may affect the catabolic pattern of nutrients. Microbial structures with high *Firmicute*/*Bacteroidete* ratios in the intestinal tract effectively absorb nutrients and promote energy accumulation and fat storage (Ley, et al., 2005; Turnbaugh, et al., 2006). For instance, differences in colon bacterial abundance and bacterial metabolites have been observed between Bama mini-pigs (a fatty-type Chinese local pig strain) and Landrace pigs (a lean-type pig strain)(Jiang, et al., 2016). Whon et al. found that castrated male cattle harbored a distinct ileal microbiome dominated by the *Peptostreptococcaceae* family, which increased obesity and improved meat quality (Whon, et al., 2021). In chickens, higher *Methanobrevibacter* and lower *Mucispirillum schaedleri* abundances were significantly positively correlated with fat deposition (Wen, et al., 2019). Therefore, the livestock and poultry microbiomes could potentially influence fat deposition. Abdominal fat deposition in Chinese indigenous ducks is associated with various advantages and disadvantages. However, few studies focus on the relationship between fat deposition and gut microbiome in Chinese indigenous ducks.

In this study, we investigate the composition, diversity, and functional characteristics of the fecal microbiota from 187 indigenous ducks (10 breeds) with different abdominal fat percentages using 16S rDNA and metagenomic sequencing. This study explores the effects of the gut microbiome on abdominal fat deposition in ducks from the perspective of multi-breed Chinese indigenous ducks, providing insights for further study of the intestinal microbiome in ducks.

## Materials and methods

### Animals and sample collection

This study conformed to the guidelines for the care and use of experimental animals established by the Ministry of Science and Technology of the People’s Republic of China (approval number: 2006–398). The research protocol was reviewed and approved by the ethical committee of Jiangxi Science and Technology Normal University. We selected ten breeds of Chinese indigenous female ducks reared at the National Waterfowl Resource Bank in Quanzhou, Fujian Province. The ducks were divided into two groups according to their abdominal fat percentage (Table 1). HAF: Sansui duck (SSD, 3.26 %), Jinding duck (JDD, 2.22 %), Chaohu duck (CHD, 1.82 %), Putian black duck (PTB, 1.29 %), and Shaoxing duck (SXD, 1.07 %); LAF: Liancheng White duck (LCW, 1.00 %), Brown tsaiya (TWD, 0.78 %), Shan Partridge duck (SPD, 0.70 %), Ji 'an red duck (JRF, 0.62 %), and Mawang duck (MWD, 0.51 %). Fresh fecal samples of ducks at 80 days of age were collected for microbiome sequencing analysis, and sample details are shown in Table 1. To minimize potential variation in microbiota composition due to geographical, dietary, and other environmental factors, all the ducks in this experiment were raised on the same farm and fed the same feed without antibiotic intervention. In addition, ducks of the same breed lived in the same duck house and were subject to strict standard farm management. Once feces samples were collected into sterile Eppendorf tubes, they were immediately stored in liquid nitrogen. All samples were then stored at −80°C until DNA extraction was performed.

**Table 1:** Sample information.

Sample Type	Breed/Line	Abbreviation	Abdominal fat (%)	Number of Samples			
				16S	Total	Metagenome	Total
High abdominal fat (HAF)	Sansui duck	SSD	3.26	18	98	4	31
	Jingding duck	JDD	2.22	20		1	
	Chaohu duck	CHD	1.82	22		12	
	Putian Black duck	PTB	1.29	19		4	
	Shaoxing duck	SXD	1.07	19		10	
Low abdominal fat (LAF)	Liancheng White duck	LCW	1.00	18	89	10	63
	Brown tsaiya	TWD	0.78	11		5	
	Shan Partridge duck	SPD	0.70	20		18	
	Ji’ an Red duck	JRF	0.62	22		16	
	Mawang duck	MWD	0.51	18		14	

### DNA extraction and high-throughput sequencing

Total genomic DNA of the gut microbiota from ducks was extracted using the CTAB/SDS method. The DNA concentration and purity were evaluated on 1 % agarose gels using the Qubit® dsDNA Assay Kit in Qubit® 2.0 Flurometer (Life Technologies, CA, USA). The V4 region within the 16S rRNA gene was amplified using primer 515F (5′-GTGCCAGCMGCCGCGGTAA-3′) and 806R (5′-GGACTACHVGGGTWTCTAAT-3′). PCR reactions comprised 3 μL forward and reverse primers (2 μM), 10 μL genome DNA (1 ng/μL), and 15 μL 2 × Phusion® High-Fidelity PCR Master Mix (New England Biolabs, United States). The thermocycling conditions were as follows: initial denaturation at 98°C for 1 min, followed by 30 cycles of 98°C for 10 s, 50°C for 30 s, and 72°C for 30 s; and final extension at 72°C for 5 min. The purified PCR products were processed for sequencing library preparation using the TruSeq® DNA PCR-Free Sample Preparation Kit (Illumina, United States). The library sequencing was performed using an Illumina NovaSeq 6000 platform, and 250 bp paired-end reads were generated.

### 16S rDNA sequencing data processing

Raw amplicon reads were assigned to their own samples using a unique barcode and were merged after barcode and primer sequence removal using FLASH (V1.2.7)(Magoc and Salzberg, 2011). High-quality clean tags were obtained using QIIME (V1.9.1)(Caporaso, et al., 2010). The tags were compared with the reference database (Silva database, https:// www.arb-silva.de/) (Quast, et al., 2013) using the UCHIME algorithm (Edgar, et al., 2011) to detect and remove chimera sequences, rendering effective tags. The Uparse software (V7.0.1001) (Edgar, 2013) was used to cluster preprocessed effective tags into OTUs exhibiting 97 % similarity. Representative sequences for each OTU were screened using the Silva Database based on the Mothur algorithm to further annotate taxonomic information. OTU abundance information was normalized using a standard sequence number corresponding to the sample with the least sequences. Subsequent analysis of alpha and beta diversities was performed using this normalized data.

### DNA extraction, library preparation, and metagenome sequencing

The extraction method for microorganism metagenomic DNA was the same as that used for 16S rDNA, and its quality was determined by agarose gel electrophoresis and NanoDrop2000. The purified PCR products were processed for sequencing library preparation using the TruSeq® DNA PCR-Free Sample Preparation Kit (Illumina, United States). The library sequencing was performed using an Illumina NovaSeq 6000 platform, and 250 bp paired-end reads were generated. Eligible DNA of approximately 350 bp in size was used to create an Illumina DNA library using the NEBNextR UItraTM DNA Library Preparation Kit (NEB, USA), and paired-end reads were sequenced using the Illumina PE150 strategy.

### Metagenomic assembly

The raw data from the Illumina HiSeq sequencing platform were processed with quality control, including low-quality read and adapter sequence removal, using Readfq (V8, https://github.com/cjfields/readfq). Bowtie2 software (version 2.2.4) was used to filter contaminated reads from the host and obtain effective data for subsequent analysis (Langmead and Salzberg, 2012). Furthermore, single samples were assembled using SOAP denovo software (V2.04, http://soap.genomics.org.cn/soapdenovo.html) and interrupted from the N connection to obtain N-free Scaftigs. Unused PE reads were generated by comparing Clean Data to Scaftigs using Bowtie2 software. To maximize data usage, the unused reads were combined, and mixed assembly was performed using SOAP denovo (V2.04) /MEGAHIT (v1.0.4-beta) (Li, et al., 2015). Finally, assembly parameters were the same as those of the single sample, while the fragments > 500 bp in all of Scaftigs were used for further analysis.

### Gene Prediction, Taxonomy, and Function Annotation

We used MetaGeneMark (V2.10, http://topaz.gatech.edu/GeneMark/) to predict open reading frames (ORFs) from the Scaftigs (> 500 bp) assembled from each sample as well as the Scaftigs from the mixed assembly (Zhu, et al., 2010). ORFs with length <100 bp were filtered out. CD-HIT software (V4.5.8, http://www.bioinformatics.org/cd-hit) was used to reduce sequence redundancy and obtain a non-redundant gene catalog (Fu, et al., 2012; Li and Godzik, 2006). DIAMOND software (V0.7.9, https://github.com/bbuchfink/diamond/) (Buchfink, et al., 2015) was used to blast the unigenes to the NCBI NR (Version 2018-01-02, https://www.ncbi.nlm.nih.gov/), KEGG (Version 2018-01-01, http://www.kegg.jp/kegg/) (Kanehisa, et al., 2006, 2014), and CAZy databases (Version 201801, http://www.cazy.org/) (Cantarel, et al., 2009). The lowest common ancestor (LCA) algorithm in MEGAN software (Huson, et al., 2011) was used to identify bacterial taxa.

### Statistical analyses and visualization

To interpret the data, we plotted the rarefaction and species accumulation curves of the gut microbiota for each duck breed. Venn/Upset diagrams were used to visualize the OTU, genus, and function distribution in the gut of ducks. The observed-species and Shannon indices were used to assess the α diversity of the sample microbiome, including non-redundant genes, OTUs, genus, and function. Next, we used PCoA based on Bray–Curtis dissimilarities to display differences in the structure and function of gut microbiota. The significant changes in community structure and function were evaluated using Wilcoxon rank sum nonparametric test (Hothorn, et al., 2008), along with analysis of similarities (ANOSIM) (Chapman and Underwood, 1999). In addition, the phyla and genera distribution was constructed with bar plots included. To identify the different microbiota and biomarkers in two groups, T test and linear discriminant analysis effect size analysis (Segata, et al., 2011) were used to identify microbial phyla and genera with significant differences in the gut of ducks with different abdominal fat ratios. The Benjamini Hochberg false discovery rate (FDR) was used to correct the p value. Finally, we used STAMP software (Parks, et al., 2014) to identify functions with significant differences in the intestines of domestic ducks with different abdominal fat ratios, including Kyoto Encyclopedia of Genes and Genomes (KEGG) secondary and KO functions, and explain the relationship between intestinal microbial function and different abdominal fat percentages in ducks.

## Results

### Fecal microbiome data of the Chinese indigenous duck

To investigate the relationship between the gut microbiome and abdominal fat accumulation, we analyzed fecal samples from 187 Chinese indigenous ducks classified into high (HAF) and low (LAF) abdominal fat groups (Table 1). Microbiome data (965 gb) was obtained by high-throughput sequencing, of which clean data (778 gb) was generated by quality control to exclude low-quality reads, adapters, or host contaminants from subsequent analyses. Based on the assembled contigs with an N50 contig length of 2.14 kb, we identified 3.58 million non-redundant genes with an average open reading frame (ORF) length of 641 bp (Table S1).

**Fig. 1:**
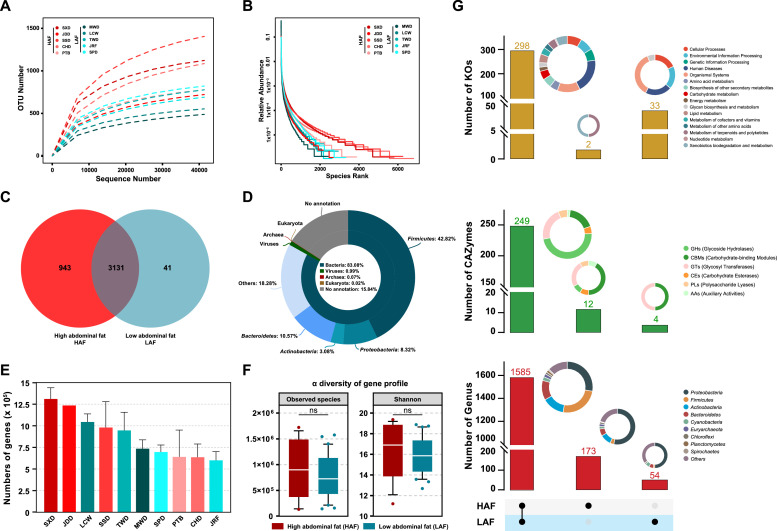
**Chinese indigenous duck fecal microbial gene catalog. (A)** Rarefaction curves and (**B**) rank abundance curves of detected operational taxonomic units (OTUs) from the whole set of 187 samples (10 breeds). (**C**) Venn diagram of common/unique OTUs in ducks with different abdominal fat ratios. (**D**) Taxonomic annotation of the Chinese indigenous duck fecal gene catalog at the superkingdom and phylum levels. (**E**) Samples of each breed were clustered to yield a set of corresponding gene catalogs. (**F**) Chinese indigenous duck fecal microbial α diversity at gene levels. (**G**) Upset plot of the levels of functional/microbial modules (KEGG orthologies [KOs], carbohydrate-active enzymes (CAZymes), and genera) of the Chinese indigenous duck fecal microbiome across different abdominal fat ratios. The panel shows sets included in the intersection and independent sites, and the bar or pie charts show the categories of the functional modules in these sets. The major enriched categories are depicted in the legend. GH, glycoside hydrolases; GT, glycosyl transferases; CE, carbohydrate esterases; PL, polysaccharide lyases; CBM, carbohydrate-binding modules; AA, auxiliary activities. (∗*p* < 0.05; ∗∗ *p* < 0.01; ∗∗∗ *p* < 0.001 for Kruskal–Wallis test and Dunn's test).

The rarefaction and species accumulation curves of all samples approached saturation, indicating that as the sample sizes increased, new microbes did not occur. The rank abundance curves of breeds and groups indicated species high abundance and uniform distribution (Fig 1A-B and S1). Notably, HAF with rich microbiomes included nearly the entire composition of that in the LAF (Fig 1C). However, according to currently available databases, only 84.16 % of the genes in the duck gut catalog were taxonomically classified at the kingdom level, among which bacteria accounted for 98.72 % of the classified genes, with the remaining genes originating from viruses, archaea, and eukarya. More than 81.72 % of the bacterial genes were from the four top phyla, including Firmicutes, Actinobacteria, Bacteroidetes, and Proteobacteria (Fig. 1D). To examine the association between functional and compositional features of the microbial communities and the host species, we constructed a representative gut microbial gene catalog for each of the ten duck breeds, spanning 0.5–1.5 million genes (Fig 1E). The microbial gene profile of HAF had higher α diversity, and there was no significant difference compared with LAF(Fig 1F). Moreover, we explored shared/unique microbial genera, Kyoto Encyclopedia of Genes and Genomes (KEGG) orthologs (Kos), and carbohydrate-active enzymes (CAZymes) in the intestinal microbiome of ducks with different abdominal fat percentages (Fig 1G). The HAF microbial genera and CAZymes exhibited higher diversity than those in LAF, except KOs. The HAF genera exhibited notable richness, especially *Firmicutes,* and *Actinobacteria* Both groups of unique microbial genera contain *Proteobacteria* and *Bacteroidetes*. In addition, the HAF fecal microbiota contains more varieties and numbers of unique CAZymes than LAF. Notably, the microbes in LAF exhibited specific KOs, which contained many human diseases and environmental information processing, while unique KOs in HAF were related to metabolism. These results suggested that the HAF microbiome might have more abundant microbiota than LAF, which were particularly prominent in metabolism-related functions. These findings highlight the importance of gut microbes and their functions for host-specific phenotypes.

### Diversity and composition of the fecal microbiome

**Fig. 2:**
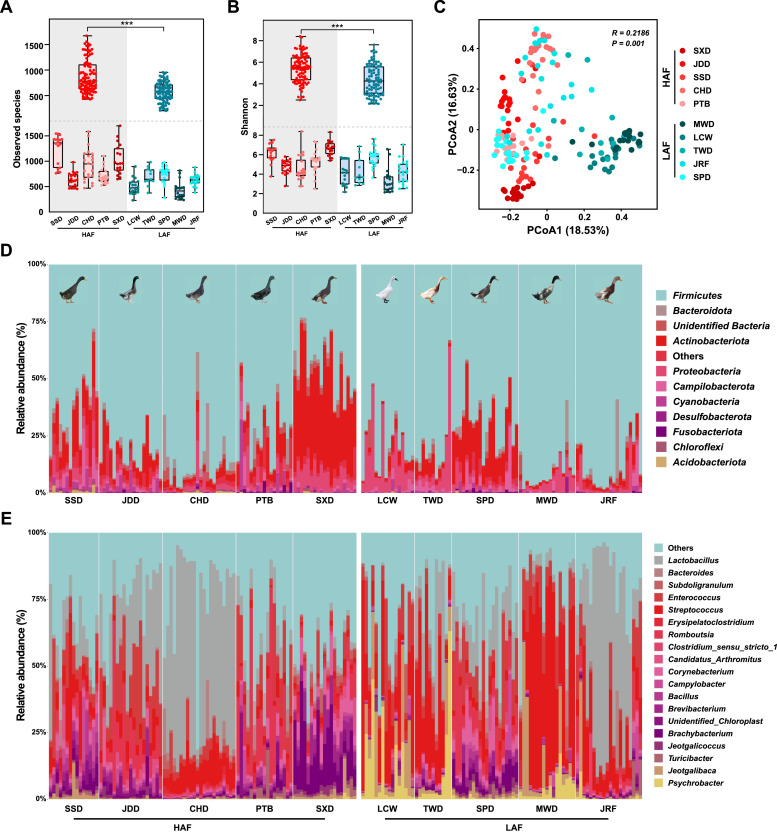
**Community structure of fecal microbiome in Chinese indigenous ducks with different abdominal fat ratios. (A)** The fecal microbial richness and (B) diversity indices of ducks with different abdominal fat ratios based on operational taxonomic unit (OTU) level. (**C**) The principal coordinate analysis (PCoA) plot based on Bray–Curtis dissimilarities and relative abundances at the OTU level. The average relative abundances of the most prevalent bacterial (**D**) phylum and (**E**) genus in each sample type plotted for samples from different abdominal fat ratios in ducks.

To determine the relationship between the gut microbiota and abdominal fat accumulation in ducks, we used the 16S data to explore the diversity, structure, and composition of the intestinal microbiota of ducks with different abdominal fat percentages. We evaluated the intestinal microbial diversity index of each duck breed based on the operational taxonomic unit (OTU) relative abundance (Table S2). Fecal microbiota OTU richness in HAFs was significantly higher than that in LAF, while the variation within the group fluctuated notably. Furthermore, microbial diversity in HAF was significantly higher than that in LAF (Fig 2A-B). Principal coordinate analysis (PCoA) showed significant changes in the microbial structure between HAF and LAF (Fig 2C). The metagenomic sequencing results also showed a consistent trend (Fig S2).

The major phyla and genera were selected to evaluate duck gut microbial compositions. *Firmicutes, Proteobacteria, Actinobacteria,* and *Bacteroidetes,* represented the main microbial phyla, accounting for > 80 % of bacteria. *Actinobacteria* were enriched in HAF, while *Proteobacteria* were decreased. Moreover, *Bacteroidetes* and *Firmicutes* were distributed evenly (Fig 2D and Table S3). At the genera level, *Lactobacillus* and *Romboutsia* were associated with HAF, while *Streptococcus, Jeotgalibaca, Psychrobacter,* and *Brevibacterium* were highly abundant in LAF. In addition, *Enterococcus* was present in all samples (Fig 2E and Table S4). These results were highly consistent with the taxonomic annotation of the metagenome (Fig S3 and Table S5, S6).

### Fecal microbial community characteristics of ducks with different abdominal fat

**Fig. 3:**
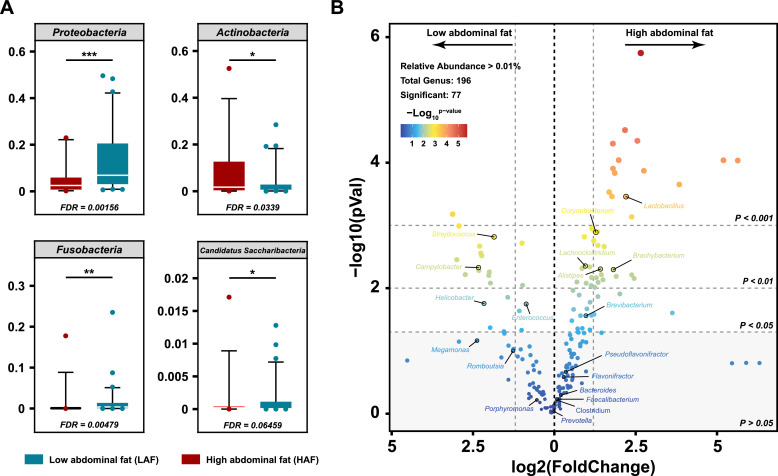
**Fecal microbial characterization of Chinese indigenous ducks with different abdominal fat ratios.** (**A**) Boxplot of differences in the relative abundance of major microbial phyla between the high/low abdominal fat ratio ducks. (**B**) Volcano plots revealed significantly different genera with relative abundance > 0.01 % between the high/low abdominal fat ratio ducks. (∗*p* < 0.05; ∗∗ *p* < 0.01; ∗∗∗ *p* < 0.001 for Student's t-test, Benjamini–Hochberg false discovery rate [FDR] obtained q value).

We performed shotgun metagenomic sequencing, which was considered to have the best resolution for genera, to characterize the microbial community features of ducks with different abdominal fat ratios. Four major phyla (relative abundance > 0.1 %) with significant differences were identified using the Wilcox test. Actinobacteria was significantly enriched in HAF, while Proteobacteria, Fusobacteria*,* Candidatus, and Saccharobacteria had a higher abundance in LAF than HAF. In addition, *Firmicutes* and *Bacteroidetes* (associated with strong catabolism and nutrient absorption) were highly abundant in HAF, but this finding was not significant (Fig 3A and Table 2). Simultaneously, the microbial genera with a relative abundance of > 0.01 % were selected to analyze significant abundance differences. A $\log_{2}\text{FC}$ was calculated to map a volcano plot labeled with the top 20 abundant genera. In total, 77 significantly different genera were identified from 196 microbial genera, with a relative abundance of > 0.01 %. Notably, the genera *Lactobacillus, Corynebacterium, Lachnoclostridium, Brachybacterium, Alistipes,* and *Brevibacterium* were significantly enriched in the HAF gut, whereas *Streptococcus, Campylobacter, Helicobacter,* and *Enterococcus* significantly enriched in the LAF gut (Fig 3B). In addition, 498 genera with significant differences among the total 2574 microbial genera were identified. When the effect size of “Difference between proportions” and “Ratio of proportions” were set to > 0.1, the 20 most representative microbial genera were identified (Table S7). The *Lactobacillus, Brachybacterium, Alistipes, Paracoccus, Corynebacterium, Erysipelatoclostridium,* and *Lachnoclostridium* abundances in the HAF gut were significantly higher than those in LAF, while the *Streptococcus, Campylobacter, Escherichia,* and *Gallibacterium* abundances showed an opposite trend (Fig S5).

**Table 2:** The relative abundances of major phylum at different abdominal fat percentages (average relative abundance > 0.1 %).

***Phylum* (relative abundance)**	**High abdominal fat (HAF)**			**Low abdominal fat (LAF)**			**log2FC(LAF/HAF)**	**p**	**FDR**
	**Mean %**	**Stderr %**	**Variance %**	**Mean %**	**Stderr %**	**Variance %**			
Firmicutes	53.1814	4.5294	6.3597	48.6851	2.5328	4.0414	0.1274	0.4166	0.6250
***Bacteroidetes***	15.3856	2.7260	2.3036	12.6792	1.7613	1.9543	0.2791	0.6294	0.7275
***Actinobacteria***	7.6759	2.0744	1.3340	2.9582	0.6951	0.3044	1.3756	0.0113	0.0339
***Proteobacteria***	4.6797	1.0893	0.3678	13.9083	1.7608	1.9533	−1.5714	0.0002	0.0016
***Fusobacteria***	0.8156	0.5745	0.1023	1.4198	0.4104	0.1061	−0.7998	0.0011	0.0048
***Tenericutes***	0.2811	0.0639	0.0013	0.2350	0.0463	0.0014	0.2581	0.8469	0.8469
***Spirochaetes***	0.2368	0.0706	0.0015	0.5232	0.1643	0.0170	−1.1436	0.0588	0.1058
***Deferribacteres***	0.1275	0.0273	0.0002	0.1966	0.0477	0.0014	−0.6250	0.6467	0.7275
***Candidatus Saccharibacteria***	0.0963	0.0552	0.0009	0.1137	0.0307	0.0006	−0.2389	0.0287	0.0646

**Fig. 4:**
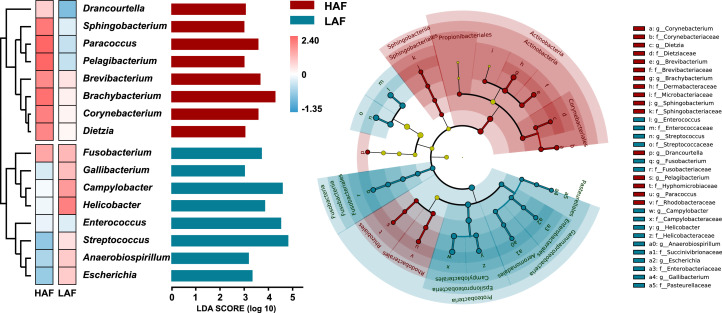
**Biomarkers identification of Chinese indigenous ducks with different abdominal fat ratios.** The biomarkers in Chinese indigenous ducks fecal microbiota determined by linear discriminant analysis effect size (LEfSe) among the high/low abdominal fat ratio ducks. (Kruskal–Wallis sum-rank test and Wilcoxon rank sum test’s p value < 0.05, and linear discriminant analysis [LDA] Score > 3).

Linear discriminant analysis effect size (LEfSe) was performed to identify specific taxa that consistently varied in abundance across ducks with different abdominal fat ratios. In total, 16 specific biomarkers for abdominal fat were identified in the two groups (Fig 4 and Table S8). *Drancourtella, Sphingobacterium, Paracoccus, Pelagibacterium, Brevibacterium, Brachybacterium, CorynebacteriumI*, and *Dietzia* served as biomarkers in HAF, while *Fusobacterium, Gallibacterium, Campylobacter, Helicobacter, Enterococcus, Streptococcus, Anaerobiospirillum*, and *Escherichia* served as biomarkers in LAF. Additionally, *Actinobacteria* was a biomarker at the microbial phylum level in HAF, while *Proteobacteria* and *Fusobacteria* were biomarkers in LAF. All of these significantly different microorganisms could be verified and repeated.

### Functional characteristics of fecal microbiota

**Fig. 5:**
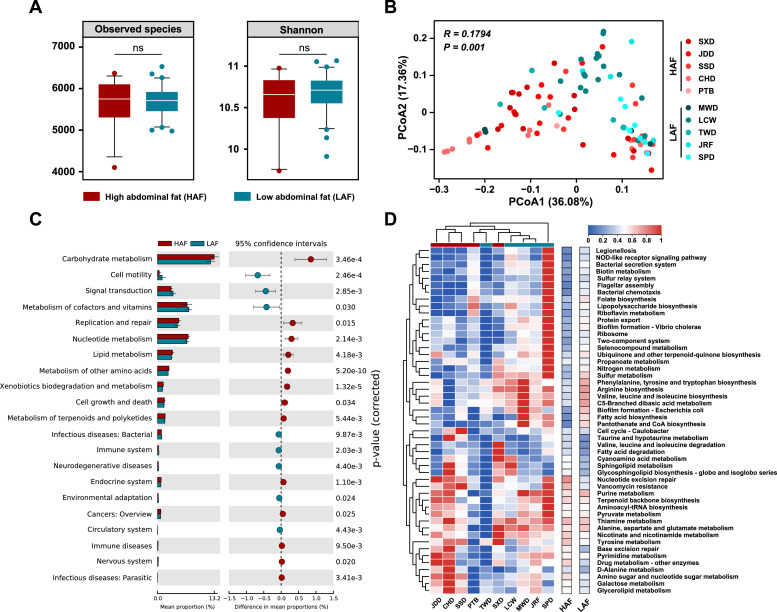
**Functional characterization of Chinese indigenous ducks with different abdominal fat ratios.** (**A**) The α diversity of microbial function in ducks with different abdominal fat ratios based on KEGG orthology (KO) catalog. (∗*p* < 0.05; ∗∗ *p* < 0.01; ∗∗∗ *p* < 0.001 for Student's t-test). (**B**) The principal coordinate analysis (PCoA) plot based on Bray–Curtis dissimilarities and relative abundances at the KO level. The significant changes of (**C**) KEGG secondary and (**D**) KO pathways in each sample type plotted for samples from the different abdominal fat ratio duck using Student's t-test.

To understand the potential relationship between intestinal microbial function and abdominal fat deposition in ducks, we annotated the gut microbial functional genes in ducks using KEGG-based metagenomic sequencing. We also evaluated the KO richness and diversity based on microbial functional abundance, along with the similarity distance of microbial functions. Our results indicated no significant difference in microbial functional diversity of ducks with different abdominal fat percentages (Fig 5A). The PCoA of microbial function showed that the functional characteristics of intestinal microbiota were separate for HAF and LAF (Fig. 5B). Twenty-one KEGG secondary metabolic pathways with significant differences between HAF and LAF were identified (Table S9). The HAF microbiota was mainly involved in carbohydrate metabolism, replication, repair, and nucleotide, lipid, amino acid, xenobiotic, terpenoid, and polyketide metabolism. Additionally, signal transduction, cofactor and vitamin metabolism, and infectious disease (bacterial and environmental adaptation) were enriched in LAF (Fig. 5C). Moreover, a total of 126 KO pathways were significantly enriched in the duck gut with different abdominal fat percentages (Table S10). The top 50 different microbial functions were selected for cluster analysis. In HAF, amino and nucleotide sugar, galactose, pyrimidine, and purine metabolism, amino acid degradation, and nucleotide excision repair had a higher abundance than in LAF. Conversely, lipopolysaccharide, branched and aromatic amino acids, folate biosynthesis and flagellar assembly, bacterial chemotaxis, ribosome, two-component system, and bacterial secretion were significantly enriched in LAF (Fig. 5D and S6).

## Discussion

Poultry meat products are a main source of protein for humans globally. Meat quality, especially in ducks, is important for consumers and livestock husbandry (Joo, et al., 2013). While considering the economic value, intensive artificial farming ignores the critical factor of meat quality. Fat deposition is a key factor that affects meat quality (Hausman, et al., 2009). Considering age, sex, nutrition, and genetics, the gut microbiome is also an important factor that affects fat deposition (Zheng, et al., 2022). Several studies in livestock and poultry suggest an association between gut microbiome and fat deposition (Chen, et al., 2021; Lei, et al., 2022; Qi, et al., 2019), but few studies consider Chinese indigenous ducks.

In this study, 187 fecal samples were collected from ducks with the unique ability to deposit abdominal fat. These samples were divided into HAF and LAF and sequenced to explore the gut microbiome characteristics using 16S rDNA and metagenomic sequencing. We aimed to elucidate the relationship between gut microbiota and abdominal fat deposition in ducks. The two microbiome strategies consistently showed that the fecal microbiome and α diversity of OTUs in HAF were higher than those in LAF; however, the metagenome showed the same trend without significance. Similarly, the intestinal microbial diversity of Beijing-You broilers with strong lipid deposition ability is high, and a fecal microbiota transplantation (FMT) experiment also supported this viewpoint (Lei, et al., 2022). Meat quality and lipid content of Angus steers were significantly positively correlated with the richness of the ruminal microbiome (Krause, et al., 2020). Our study revealed a significant change in the microbial community structure of ducks with different abdominal fat percentages. Similar R and significant P values were obtained, which indirectly reflected the consistency of the two sequencing strategies. In Chinese indigenous ducks, the fecal microbiota composition at the phylum level was mainly composed of Firmicutes, Bacteroides, Actinobacteria, and Proteobacteria, which is similar to that in birds, chickens, and ducks (Cao, et al., 2020; Grond, et al., 2018; Huang, et al., 2018). Moreover, our results suggested that Actinobacteria was significantly enriched in HAF, while Proteobacteria, Fusobacteria, and Saccharobacteria had higher abundance in LAF than HAF. In the Angus cattle gut, Actinobacteria was significantly positively correlated with lipid content and 12th rib fat thickness, while Proteobacteria and Saccharibacteria (formerly known as TM7) were the opposite (Krause, et al., 2020). In humans, Fusobacteria is associated with colorectal cancer (Zhang, et al., 2023), whereas its enrichment might be associated with intestinal health in ducks. In addition, Firmicutes and Bacteroides, which play a crucial role in nutrient decomposition and energy metabolism, were more abundant in HAF than LAF (not significant) (Polansky, et al., 2015). In this study, a total of 498 genera with significant differences between HAF and LAF were identified in all microbial genera detected. The *Lactobacillus, Alistipes, Corynebacterium,* and *Lachnoclostridium* genera were more abundant in the gut of HAF than in that of LAF. Notably, *Lactobacillus* is often regarded as a probiotic representative, and its metabolites increase intestinal lipid absorption and promote rapid fat accumulation in the intestinal tract of mice (Zhong, et al., 2022). These findings may suggest an important factor regarding the correlation between fat accumulation and gut microbes in HAF. In a study regarding fat deposition-associated gut microbes in pigs, *Alistipes* in the cecum were highly positively correlated with backfat thickness and intramuscular fat (Tang, et al., 2020). Notably, high *Corynebacterium* abundance is associated with *ELOVL2* (Wen, et al., 2021), which is involved in long-chain polyunsaturated fatty acid elongation and lipid synthesis (Gregory, et al., 2013; Jakobsson, et al., 2006; Pauter, et al., 2014). In a study on the relationship between gut microbes and chicken meat quality, Lei et al. found that *Lachnoclostridium* had a high relative abundance in the gut of Beijing-You broilers with a high percentage of abdominal fat. Furthermore, they found that in Arbor Acres broilers transplanted with the fecal microbiota from Beijing-You broilers, *Lachnoclostridium* was identified as the only bacteria genus that was positively associated with meat fiber diameter, body weight, and abdominal fat rate but negatively associated with drip-losing rate (Lei, et al., 2022). We found that *Streptococcus, Campylobacter, Helicobacter, Enterobacter, Gallibacterium,* and *Escherichia* were significantly enriched in LAF, and some of these genera can be considered pathogens or even biomarkers. Qi et al. reported the effect of culture patterns on pork quality and found that *Streptococcus* was significantly negatively correlated with the intramuscular fat area, while *Clostridium* was the opposite (Qi, et al., 2019). In this study, *Clostridium* had a higher abundance in HAF than in LAF (not significant). Moreover, Tang et al. reported that pigs fed fermented complete feed had better meat quality, which was indicated by a high unsaturated fatty acid content and a low average back-fat thickness. Fermented complete feed also significantly reduced the relative abundances of presumably pathogenic bacteria from the *Proteobacteria* phylum and the *Escherichia–Shigella* genus (Tang, et al., 2021). Generally, *Campylobacter, Helicobacter,* and *Escherichia* represent the major zoonotic bacterial pathogens that cause food-borne illness and death. Furthermore, these genera are generally associated with contaminated animal products (Abebe, et al., 2020; Mladenova-Hristova, et al., 2017). Many studies report that the *Gallibacterium* genus, which can colonize chickens, ducks, and birds, is an opportunistic pathogen in poultry breeding and can cause occasional diseases such as ovaritis, salpingitis, peritonitis, and enteritis (El-Adawy, et al., 2018; Kristensen, et al., 2011). These pathogens cause animal disease and pose a serious threat to human health.

Although the gut microbiota plays a crucial role in fat deposition in livestock and poultry, understanding how the gut microbiota performs its function properly is critical. In this study, metagenomics was used to characterize the intestinal microbial functional profile and explore the functional characteristics of the gut microbiome in ducks with different abdominal fat rates. No significant difference was found in the α diversity of microbial function in ducks with different abdominal fat percentages; however, a significant change in the functional structure of gut microbiota was observed. Twenty-one secondary metabolic and 126 KO pathways were significantly enriched in Chinese duck gut with different abdominal fat percentages. The gut microbiota in HAF were mainly involved in carbohydrate metabolism, nucleotide, lipid, amino acid, xenobiotics, terpenoids, and polyketide metabolism. These functions were similarly enriched in the intestines of Ningxiang piglets, which are a fatty-type Chinese indigenous pig breed (Lei, et al., 2021). Some KOs, such as glycerolipid, tyrosine, and purine metabolism, as well as sphingolipid biosynthesis, were enriched in the intestine of pigs with low lean meat percentages and in the gut of ducks with higher abdominal fat percentages (Chen, et al., 2021). In addition, signal transduction, cofactor and vitamin metabolism, and infectious disease (bacterial and environmental adaptation) were enriched in LAF. The function term of metabolism of cofactors, vitamins, and amino acids, such as arginine, were significantly more abundant in the gut microbiome of lean pigs, which was consistent with the low abdominal fat ducks (Chen, et al., 2021). Interestingly, vitamins K, D3, pantothenic acid, and biotin, which are associated with decreased common obesity (Foss, 2009), reduced inflammation (Jarvinen, et al., 2016), and increased energy expenditure and adiponectin expression (Hussein, et al., 2018), were enriched in LAF. Notably, lipopolysaccharide biosynthesis, flagellar assembly, bacterial chemotaxis, two-component systems, and bacterial secretion systems were highly enriched in fat pigs (Chen, et al., 2021), which could function to increase the capacity for energy harvest from bacteria (Alm, et al., 2006; Turnbaugh, et al., 2006), impair host gut barrier integrity, and mediate inflammation. Although contrary to the findings of this study, we suggest that the significant enrichment of these functions in the intestines of LAF might be related to the high pathogenic bacteria abundance, all of which were Gram-negative bacteria with flagella.

## Conclusion

This study revealed that the gut microbiome of ducks with different abdominal fat ratios had significant differences. The increase of gut bacteria involved in fat accumulation and the decrease of some pathogenic bacteria may be the main factors that promote abdominal fat deposition. In addition, the enrichment of various metabolism pathways promotes nutrient catabolism and provides energy for host fat accumulation. Conversely, cofactor and vitamin metabolism-related functions and bacterial components increase energy expenditure and compete for host energy. This study provides a valuable reference for screening and identification of abdominal fat accumulation-related gut microbiota in Chinese indigenous ducks.

Fig. S1. Rarefaction and rank abundance curves and species accumulation boxplot of detected operational taxonomic units (OTUs) from the high/low abdominal fat ratio ducks.

Fig. S2. Fecal microbial diversity of the high/low abdominal fat ratio ducks based on metagenomic sequencing. (A) The fecal microbial α diversity of ducks with different abdominal fat ratios based on genus level. (B) The principal coordinate analysis (PCoA) plot based on Bray–Curtis dissimilarities and relative abundances at the genus level.

Fig. S3. Microbial community compositions of 10 Chinese indigenous duck breeds across 94 ducks fecal samples based on metagenomic sequencing.

Fig. S4. Differences in major microbial phyla between the high/low abdominal fat ratio ducks.

Fig. S5. The top 20 most representative microbial genera with significant changes between the high/low abdominal fat ratio ducks.

Fig. S6. The most representative microbial function with significant change between the high/low abdominal fat ratio ducks based on Kyoto Encyclopedia of Genes and Genomes orthology (KO) pathways.

## Declaration of competing interest

The authors declare that they have no competing interests.

## Appendix Supplementary materials

Image, application 1
